# ERBB2 as a prognostic biomarker correlates with immune infiltrates in papillary thyroid cancer

**DOI:** 10.3389/fgene.2022.966365

**Published:** 2022-11-09

**Authors:** Yuchen Jin, Xian Qiu, Ziyan He, JunYao Wang, Ri Sa, Libo Chen

**Affiliations:** Department of Nuclear Medicine, Shanghai Jiao Tong University Affiliated Sixth People’s Hospital, Shanghai, China

**Keywords:** ERBB2, prognosis, biomarker, immune microenviroment, papillary thyroid cancer

## Abstract

Epidermal growth factor receptor 2 (*ERBB2*) is commonly over-expressed in advanced or metastatic tissues of papillary thyroid cancer (PTC) with poor prognosis, while it remains unknown whether *ERBB2* plays a role in the progression of PTC. Thus, we analyzed the data derived from online repositories, including TCGA, KEGG, GO, GeneMANIA, and STRING, to explore the relationship between *ERBB2* expression and prognosis, tumor phenotypes of interest, and immune infiltrates in PTC. Compared to normal thyroid tissue, *ERBB2* was up-regulated in PTC samples (*p* < 0.001); In comparison with the group with low expression of *ERBB2*, the group with high expression of *ERBB2* had poorer progression-free interval in stage III/IV patients (*p* = 0.008) and patients aged >45 years (*p* = 0.019). The up-regulated *ERBB2* was associated with iodine metabolism dysfunction, proliferation, metastasis, angiogenesis, and drug resistance. The expression of *ERBB2* negatively correlated with enrichment scores of B cells (r = −0.176, *p* < 0.001), CD8^+^ T cells (r = −0.160, *p* < 0.001), cytotoxic cells (r = −0.219, *p* < 0.001), NK CD56dim cells (r = −0.218, *p* < 0.001), plasmacytoid dendritic cells (r = −0.267, *p* < 0.001), T cells (r = −0.164, *p* < 0.001), T follicular helper cells (r = −0.111, *p =* 0.012), gamma delta T cells (r = −0.105, *p =* 0.017), and regulatory T cells (r = −0.125, *p* = 0.005). In conclusion, *ERBB2* may serve as a prognostic biomarker and an immunotherapeutic target in PTC, deserving further exploration.

## Introduction

Thyroid cancer is one of the most common endocrine neoplasms, and the incidence rate is overgrowing, with 53,815 new cases expected in the US (Siegel et al., 2021) and 224,023 in China (Xia et al., 2022) in 2022. Papillary thyroid cancer (PTC) is the most widely recognized thyroid tumor, accounting for around 84% of all thyroid cancer patients (Fagin and Wells, 2016). Alarmingly, the incidence of invasive and metastatic PTC has risen 1.5–5-fold over the past 30 years (Lim et al., 2017). Within 10 years after initial surgery and radioiodine therapy, approximately 1/10 to 1/5 of PTCs developed local recurrence and distant metastasis; Once distant metastases of PTC occurred, the 5-year survival rate of patients would decrease from 98% to 63–75% (Cancer Facts 2022). The high rates of recurrence, metastasis, and radioiodine refractoriness have become the key bottleneck, stymying the cure of PTC (Jin et al., 2018).

Epidermal growth factor receptor 2 (*ERBB2*), a well-known oncogene of multiple cancers, such as breast cancer and ovarian cancer (Baxevanis et al., 2004), was found to be firmly connected with cancer occurrence, proliferation, metastasis, drug resistance, immune escape and poor prognosis (Dawood et al., 2009; Bates et al., 2018). Owe to the research on *ERBB2*, multiple diagnostic and therapeutic methods were established for tumor management (Mitri et al., 2012). In thyroid cancer, however, the deep-going research on *ERBB2* is limited. Although *ERBB2* is over-expressed in progressive and metastatic PTC cases (Kremser et al., 2003), and high expression of *ERBB2* was found to be responsible for resistance to mitogen-activated extracellular signal-regulated kinase inhibitors (MEKi) (Montero-Conde et al., 2013), the *ERBB2* upregulation-related unfavorable phenotypes, such as tumor occurrence, proliferation, metastasis, and poor progression-free interval (PFI), have not been comprehensively explored in PTC, and the relations between *ERBB2* expression and the immune microenvironment in PTC remains unknown.

Given that the application of *ERBB2*-related management approaches, such as imaging probes and immunotherapeutic drugs, is limited by lacking knowledge of the association between *ERBB2* expression and oncological features of PTC; we, therefore, conducted the bioinformatic study with integrated data from online repositories, including TCGA, KEGG, GO, GeneMANIA, and STRING to explore the potency and translational value of *ERBB2* as a prognostic biomarker and immunotherapeutic target for PTC management.

## Materials and methods

### Data collection

The mRNA data and clinical characteristics of PTC patients were downloaded from the TCGA database (https://www.cancer.gov/tcga/), an open data portal that compiled clinical information and RNA-Seq data of 33 cancers. The thyroid cancer (THCA) project of the TCGA database includes 568 samples (510 PTC and 58 paracancerous normal samples). Clinical data collection for the THCA project was mainly completed in 2014; thus, PTC staging was adopted in AJCC 7^th^ edition (Agrawal et al., 2014). The mRNA sequencing data of all samples were converted to the format of transcripts per million (TPM) for subsequent analysis.

### OncoPrint analysis online

OncoPrint is a method for visualizing samples by integrating gene expression heat map and gene variant distribution map. The gene expression and gene mutation data in OncoPrint analysis were derived from the TCGA-THCA dataset and analyzed online (https://www.cbioportal.org). The OncoPrint analysis was utilized to show the association between *ERBB2* expression and *BRAF* and *RAS* mutant PTC sample distribution.

### Progression-free interval analysis

The Kaplan-Meier Progression-free interval (PFI) curve compared prognosis differences between patients with high and low expression of *ERBB2*. Since the death reports of PTC in the TCGA database were few (n = 16), the clinical prognosis data included in this study were PFI. Due to the limited number of patients with distant metastases in the database (n = 9), we could not analyze PFI in the subgroup of patients with distant metastases.

### Co-expression heat map

Co-expression heat map assesses the correlation between the expression of *ERBB2* and gene sets, including the iodine metabolism-related gene set of *TSHR*, *SLC5A8*, *SLC26A4*, and *TPO* (Portulano et al., 2013), the tumor angiogenesis gene set of *VEGFA*, *FLT1*, *KDR*, *FLT4*, *PECAM1*, *VWF*, *TIE1*, *TEK*, *ANGPT1*, *ANGPT2*, *CDH5*, and *CLDN5* (Smith et al., 2010; Haibe et al., 2020), Lymph node metastasis gene set of *EVA1A*, *TIMP1*, *SERPINA1*, *FAM20A*, *FN1*, *TNC*, and *MXRA8* (Wu et al., 2021), distant metastatic gene sets of *MMP2*, *PTTG1*, *VEGFC*, *CXCR4*, and *FGF2*, tumor cell proliferation set of *MKI67*, *PCNA*, and *MCM2*) (Liang et al., 2011), and MEKi resistance marker gene set of *SPRY2*, *SPRY4*, *ETV4*, *ETV5*, *DUSP4*, *DUSP6*, *CCND1*, *EPHA2*, and *EPHA4* (Mazzoni et al., 2019; Degirmenci et al., 2021).

### Analysis of immune infiltrates

The enrichment scores of 24 immune cells were based on the reported literature (Bindea et al., 2013). The correlation analysis between *ERBB2* expression and enrichment scores of immune infiltration was tested with a single sample gene set enrichment analysis (ssGSEA) with R packages of clusterProfiler (version 3.8.0).

### Analysis of ERBB2-related differentially expressed genes (DEGs)

To further understand the role of *ERBB2* in the progression of PTC, we screened the DEGs between samples with high and low expression of *ERBB2*. We applied Log_2_(fold change) > 1.5 and adjusted *p* < 0.001 to select DEGs. The analysis is performed using the R package of DESeq2 (version 3.8). All the DEGs were graphed in a volcano plot.

### Enrichment analysis of KEGG and GO terms

GO analysis is a widely applied bioinformatics tool for determining *ERBB2*-related biological processes, cellular components, and molecular functions. *KEGG* analysis is used to analyze *ERBB2*- related signal path changes. We applied GO and *KEGG* to analyze the biological function of *ERBB2* in PTC. To understand how *ERBB2* is involved in tumorigenesis, we used *KEGG* and GO online tools to analyze the signaling pathways and gene functions in which *ERBB2*-related DEGs participated. Gene set enrichment analysis (GSEA) with R package clusterProfiler (version 3.8.0) evaluated the *ERBB2*- related DEGs contributing to annotated gene functions, cell phenotypes, signal pathways, and diseases.

### GeneMANIA and string analysis online

The network of gene-gene relations was constructed with the online tool GeneMANIA (https://genemania.org/). The website extensively integrated data on gene-gene interactions, gene co-expression networks, and gene function enrichment. To understand the *ERBB2*-involved mechanisms, the STRING tool (version 11.5, https://string-db.org/) was utilized to analyze the protein-protein interaction (PPI) network of corresponding proteins of *ERBB2*-related DEGs. The PPI network was displayed with Cytoscape software (version 3.9.1).

### Statistical analysis

All statistical analyses and graphs were performed using R software (version 3.6.3). The *ERBB2* expression in unpaired and paired samples was analyzed with Wilcoxon rank-sum test and Wilcoxon signed-rank test, respectively. Kruskal-Wallis test was tested to assess the relationship between clinical/cytogenetic features and *ERBB2* expression. The differences in enrichment scores of immune infiltrate were analyzed using the Wilcoxon rank sum tests. The Spearman statistical method was adopted for the correlation analysis between ERBB2 expression and the gene expression of interest or enrichment scores of 24 immune cells. Hazard ratio, log-rank tests, and the Kaplan-Meier curve were applied to assess the role of *ERBB2* as a prognostic biomarker. *P* < 0.05 was considered statistically significant.

## Results

### Aberrantly upregulated *ERBB2* expression in PTC samples

**FIGURE 1:**
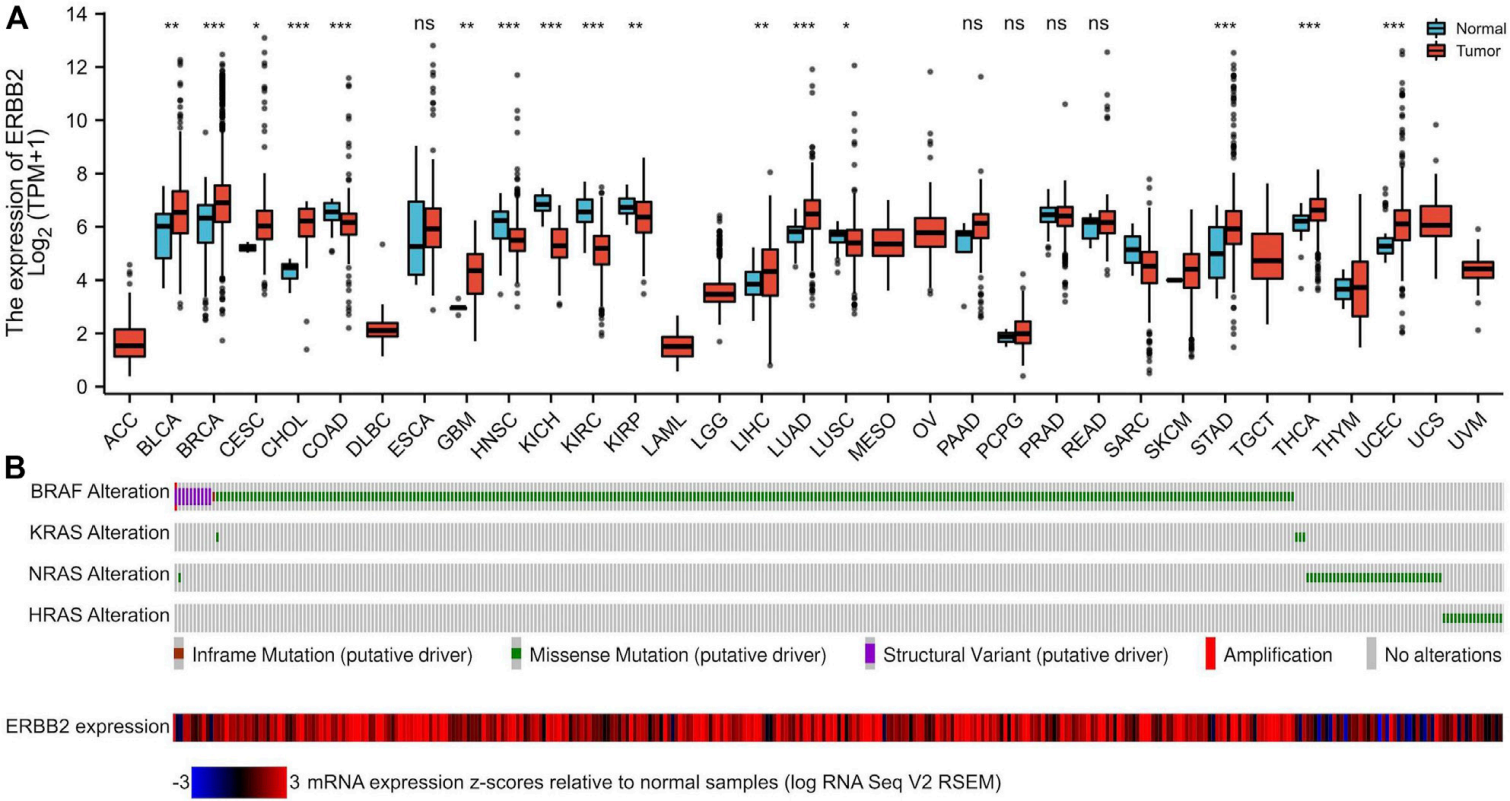
The mRNA expression of *ERBB2* in pan-cancers and tumor-adjacent normal samples. **(A)** Compared with paired normal tissues, mRNA expression of ERBB2 was upregulated in 16 cancer datasets: BLCA (*p* = 0.007), BRCA (*p* < 0.001), CESC (*p* = 0.034), CHOL (*p* < 0.001), GBM (*p* = 0.002), LIHC (*p* = 0.008), LUAD (*p* < 0.001), STAD (*p* < 0.001), and THCA (*p* < 0.001) and UCEC (*p* < 0.001). **(B)** The OncoPrint plot displays an overview of gene alterations of the *BRAF*, *KRAS*, *NRAS*, and *HRAS*, and the expression of *ERBB2* in papillary thyroid cancer samples from TCGA. ^*^ *p* < 0.05, ^**^ *p* < 0.01, ^***^ *p* < 0.001.

**FIGURE 2:**
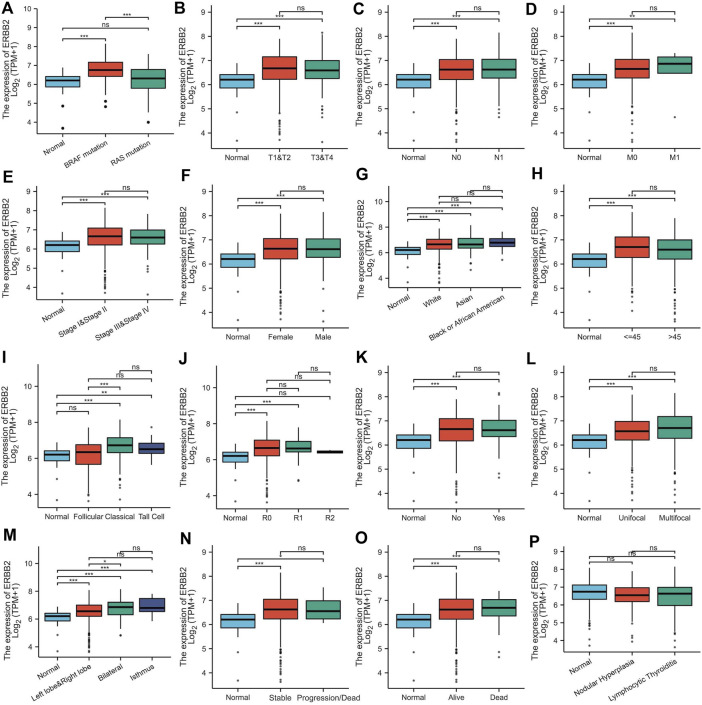
*ERBB2* expression in papillary thyroid cancer classified by characteristics: **(A)** mutation, **(B)** T stage, **(C)** N stage, **(D)** M stage, **(E)** pathological stage, **(F)** gender, **(G)** race, **(H)** age, **(I)** histological type, **(J)** residual tumor, **(K)** extrathyroidal extension, **(L)** primary tumor focus type, **(M)** tumor location, **(N)** overall survival event, **(O)** progression-free interval event, and **(P)** thyroid gland disorder history. ^*^ *p* < 0.05, ^**^ *p* < 0.01, ^***^ *p* < 0.001.

By analyzing the RNA-Seq data downloaded from TCGA data, we found that the *ERBB2* expression in PTC samples was generally higher than that in normal thyroid tissue (unpaired test: *p* < 0.001, Figure 1A; paired test: *p* < 0.001, Supplementary Figure S1). OncopPrint plot displayed that PTC tissues with high expression of *ERBB2* were mainly distributed in *BRAF* mutation samples rather than *RAS* mutation samples (Figure 1B). Subgroup analysis showed that the *ERBB2* expression in *BRAF* mutant PTC, classic PTC, and PTC with bilateral foci were significantly higher than those in corresponding subgroups of *RAS* mutant PTC (*p* < 0.001), follicular variant PTC (FVPTC) (*p* < 0.001), and PTC with unilateral foci (*p* = 0.02), respectively (Figure 2). To determine the *ERBB2*-related clinical value and the underlying mechanism, PTC samples with high and low expression of *ERBB2* were divided based on the median TPM value. The characteristics of patients with the high and low expression of *ERBB2* were summarized in Supplementary Table S1.

### Elevated *ERBB2* expression was associated with the poor prognosis

**FIGURE 3:**
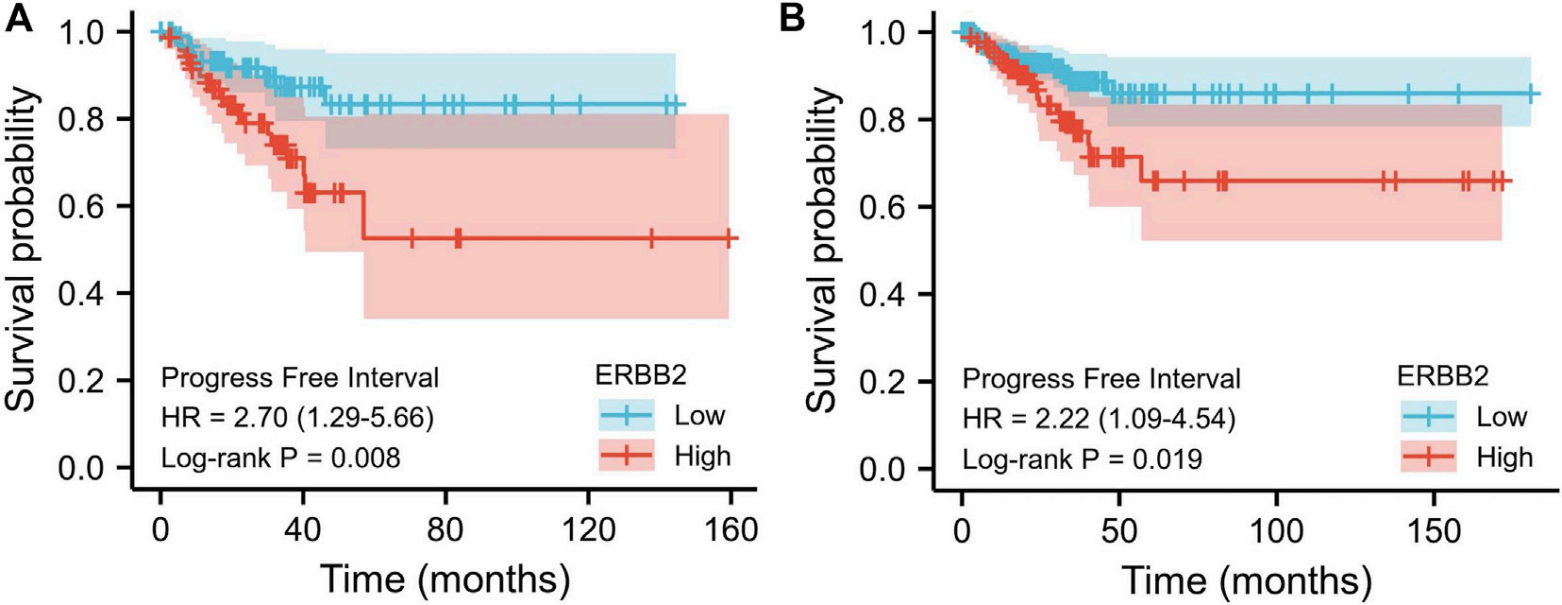
Kaplan-Meier curves of progression-free interval comparing the high and low expression of *ERBB2* in thyroid cancer patients. **(A)** In stage III/IV patients, the progression-free interval of the high *ERBB2* expression group was poorer than that of the low expression group (*p =* 0.008). **(B)** In patients aged >45 years, the progression-free survival of the high *ERBB2* expression group was poorer than that of the low expression group (*p =* 0.019).

The relations between *ERBB2* expression and PFI were analyzed using the Kaplan-Meier curve. As is shown in Figure 3, the group with high expression of *ERBB2* had a significantly poorer prognosis than that with low expression of *ERBB2* in stage III/IV patients (HR: 2.7, CI: 1.29–5.66, *p* = 0.008) and patients aged >45 years (HR: 2.22, CI: 1.09–4.54, *p* = 0.019) (Figure 3); the detailed subgroup analyses of PFI Kaplan-Meier curves were plotted in Supplementary Figures S2–4.

### Highly expressed *ERBB2* was correlated to unfavorable tumor phenotypes

**FIGURE 4:**
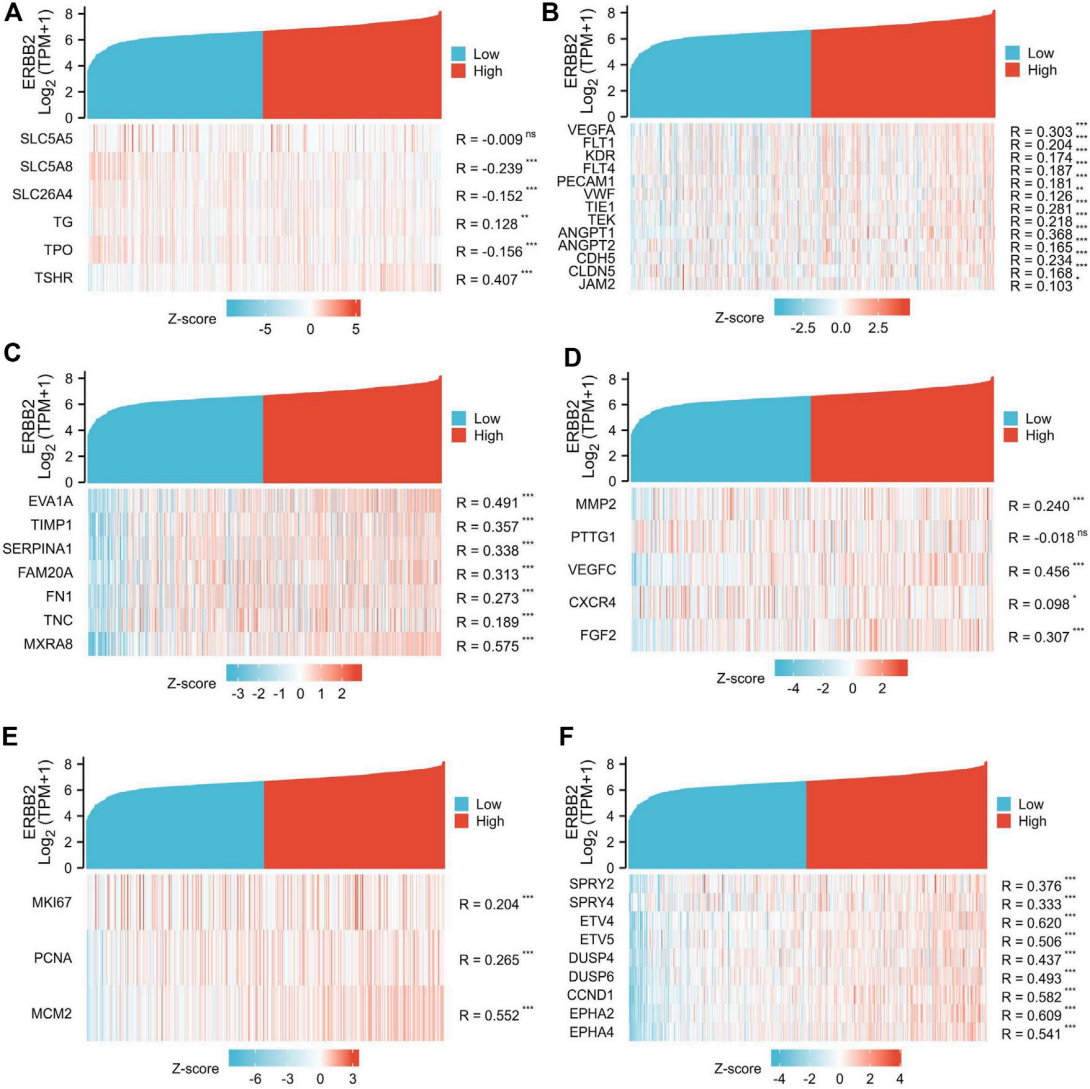
Co-expression analysis of *ERBB2* and genes related to unfavorable thyroid cancer phenotypes. **(A)** the expression of *ERBB2* is negatively correlated with iodine metabolism genes of *SLC5A8*, *SLC26A4*, and *TPO*. **(B)** the expression of *ERBB2* was positively correlated with the tumor angiogenesis gene set. **(C)** the expression of *ERBB2* is positively correlated with the gene set of lymph node metastasis. **(D)** the expression of *ERBB2* was positively correlated with the distant metastasis gene set. **(E)** the expression of *ERBB2* was positively correlated with the gene set of cell proliferation. **(F)** the expression of *ERBB2* was positively correlated with the MEK inhibitor resistance gene set. ^*^ *p <* 0.05, ^**^ *p* < 0.01, ^***^ *p* < 0.001.

The association between *ERBB2* expression and clinical phenotypes was performed with correlation analysis. The co-expression heat map showed that the expression of *ERBB2* positively correlated with *TSHR* but negatively correlated with *SLC5A8*, *SLC26A4*, and *TPO* (Figure 4A); In addition, the expression of *ERBB2* positively correlated with gene sets of tumor angiogenesis (*VEGFA*, *FLT*1, *KDR*, *FLT4*, *PECAM1*, *VWF*, *TIE1*, *TEK*, *ANGPT1*, *ANGPT2*, *CDH5*, and *CLDN5*) (Figure 4B), lymph node metastasis (Gene sets: *EVA1A*, *TIMP1*, *SERPINA1*, *FAM20A*, *FN1*, *TNC*, and *MXRA8*) (Figure 4C), distant metastases (Gene sets: *MMP2*, *VEGFC*, *CXCR4*, and *FGF2*) (Figure 4D), tumor cell proliferation (Gene sets: *MKI67*, *PCNA*, and *MCM2*) (Figure 4E), and MEKi resistance (Gene sets: *SPRY2*, *SPRY4*, *ETV4*, *ETV5*, *DUSP4*, *DUSP6*, *CCND1*, *EPHA2*, and *EPHA4*) (Figure 4F).

### Up-regulated *ERBB2* was associated with suppressed tumor-infiltration of immune cells

**FIGURE 5:**
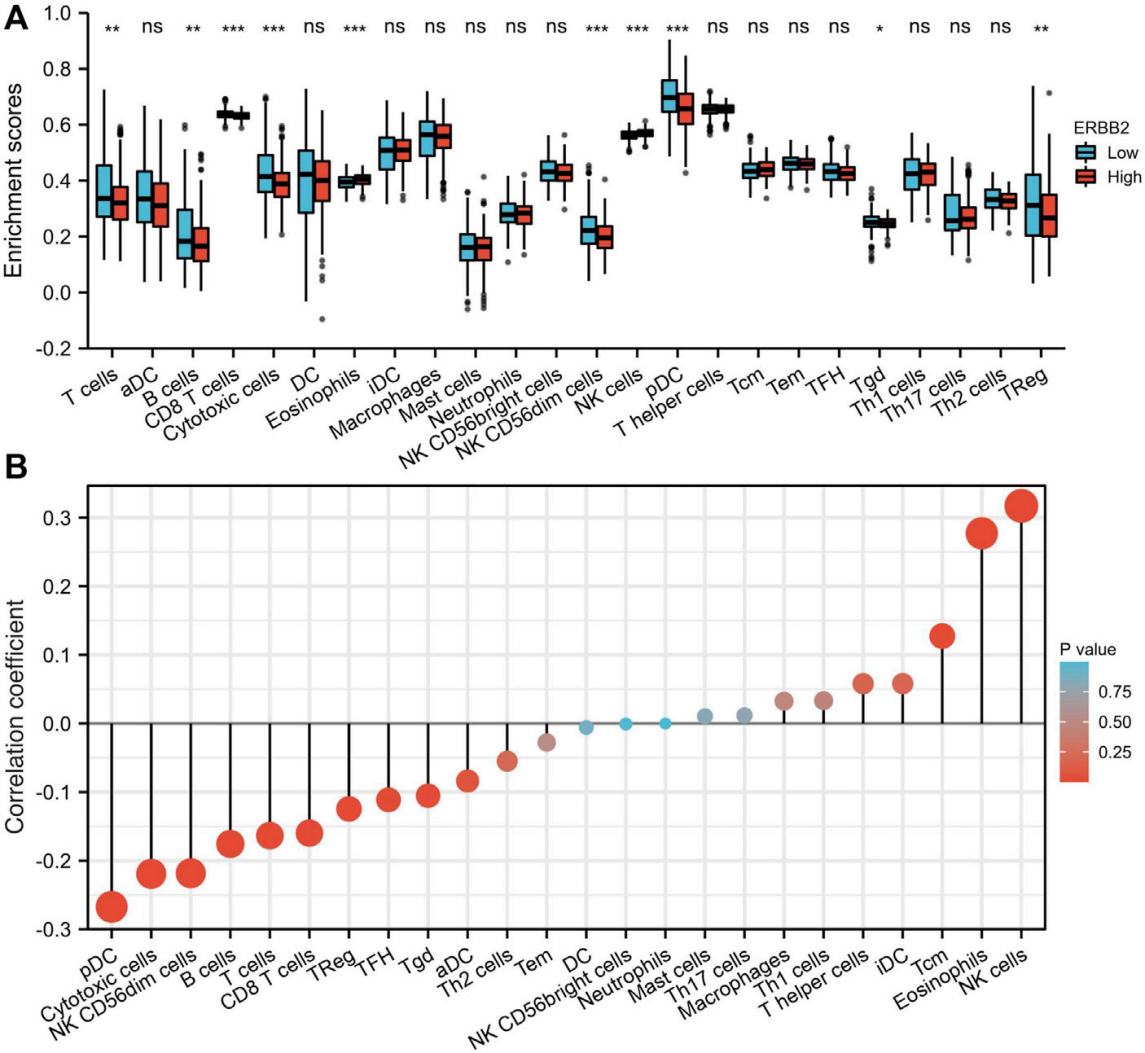
Immune cell enrichment scores in thyroid cancer tissues with different *ERBB2* expression. **(A)** The enrichment scores of T cells, B cells, CD8^+^ T cells, cytotoxic cells, NK CD56dim cells, pDCs, Tgd, and TReg in the *ERBB2* high expression group were lower than those in the *ERBB2* low expression group. **(B)** Correlation analysis between the expression of *ERBB2* and the enrichment scores of immune infiltrating cells in the tumor. *ERBB2* expression negatively correlated with the enrichment scores of CD8^+^ T cell, cytotoxic cell, NK CD56dim cell, pDC, T cell, TFH, Tgd, and TReg. DC, dendritic cell; aDC, activated dendritic cell; pDC, plasmacytoid dendritic cell; iDC, interdigitating dendritic cell; Tcm, central memory T cell; Tem, effector memory T cell; TFH, follicular helper T cell; Tgd, T gamma delta, γδ; Th1, T helper type 1; Th17, T helper type 17; Th2, T helper type 2; Treg, regulatory T cells; NK, natural killer. ^*^ *p* < 0.05, ^**^ *p* < 0.01, ^***^ *p* < 0.001.

The tumor-infiltrating immune cells were quantified by ssGSEA. Compared to the samples with low expression of *ERBB2*, the enrichment scores of T cells, CD8^+^ T cells, cytotoxic cells, NK CD56dim cells, plasmacytoid dendritic (pDC) cells, gamma delta T cells (Tgd), and regulatory T cells (TReg) were lower in samples with high expression of *ERBB2* (Figure 5A). The correlation analysis showed that *ERBB2* expression was negatively correlated with the enrichment scores of B cells, CD8^+^ T cells, cytotoxic cells, NK CD56dim cells, pDC, T cells, follicular helper T cells (TFH), Tgd cells, and TReg cells (Figure 5B, Supplementary Table S2).

### Identification of DEGs between samples with high and low expression of *ERBB2*

**FIGURE 6:**
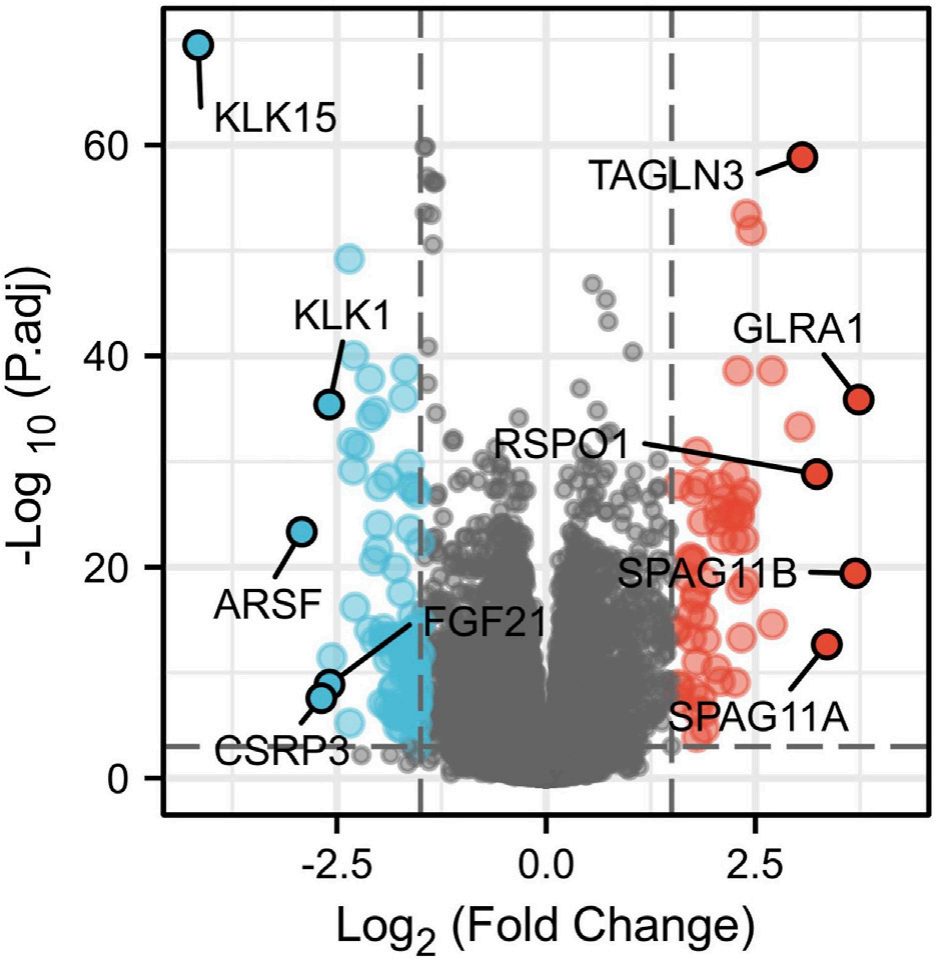
Volcano plot of differentially expressed genes (DEGs) between high and low *ERBB2* expression groups. The significantly up-regulated genes are red dots; the significantly down-regulated genes are blue dots. The dots marked with gene names are the most up-regulated five DEGs and the most down-regulated five DEGs.

A total of 146 DEGs between samples with high and low expression of *ERBB2* were yielded (Supplementary Table S3). The five most up-regulated genes and the five most downregulated genes were shown in the volcano plot (Figure 6). Spearman correlation analysis found that the expression of *ERBB2* negatively correlated with *KLK15*, *KLK1*, *ARSF*, and *FGF21*; and positively correlated with *TAGLN3*, *GLRA1, RSPO1*, *SPAG11B*, and *SPAG11A* (Supplementary Figure S5).

### *ERBB2*-related DEGs participated in the primary immunodeficiency signaling pathway

**FIGURE 7:**
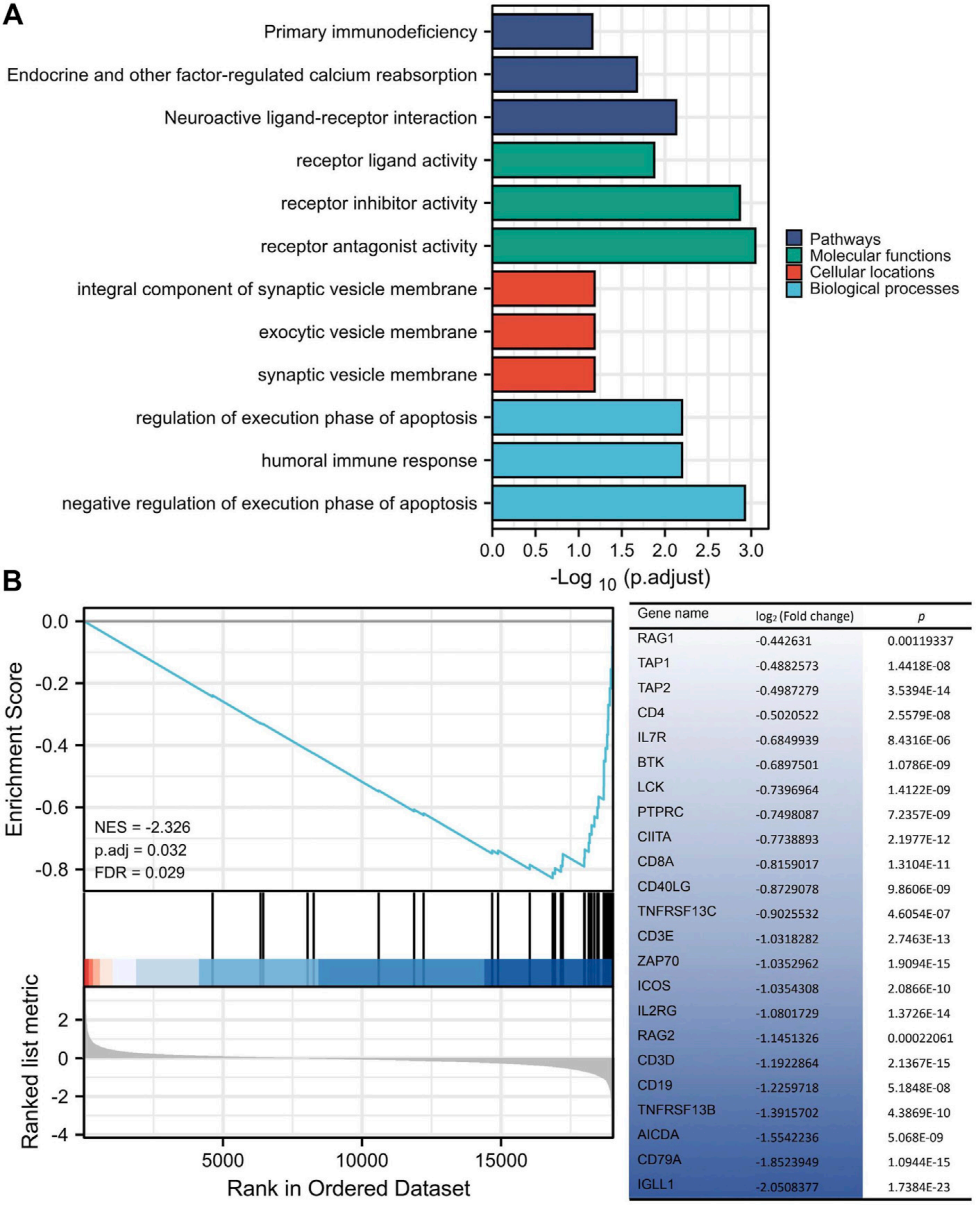
KEGG and GO enrichment analysis of differentially expressed genes between high and low expression of *ERBB2* groups. **(A)** 146 ERBB2-related differentially expressed genes were enriched in KEGG signaling pathways and GO annotations, such as the primary immunodeficiency. **(B)** GSEA analysis of 19433 protein-coding genes and 146 ERBB2-related differentially expressed genes showed that the genes involved in the primary immunodeficiency pathway were mainly 23 down-regulated genes in the *ERBB2* high expression group; Gene expression was sorted by log_2_ (fold change).

Annotations from KEGG and GO were enriched with 146 *ERBB2*-related DEGs, showing that the *ERBB2*-related DEGs were involved in the KEGG term of primary immunodeficiency and GO term of the humoral immune response, regulation of execution phase of apoptosis, and negative regulation of execution phase of apoptosis (Figure 7A). Thereinto, the majority of *ERBB2*-related DEGs (n = 23) were enriched into the primary immunodeficiency signaling pathway, attracting more attention. GSEA analysis adds more insight into the 23 *ERBB2*-related DEGs and primary immunodeficiency signaling pathway, showing that *ERBB2*-related DEGs associated with primary immunodeficiency were mainly down-regulated *ERBB2*-related DEGs, such as *CD79A*, *CD19*, *CD3D*, *CD3E*, *CD8A*, and *CD4* (Figure 7B).

### *ERBB2*-related DEGs constructed the core PPI networks

Network analysis showed that the 23 *ERBB2*-related DEGs had gene interaction and co-expression relationships with each other; the functional enrichment found that the 23 *ERBB2*-related DEGs involved in the function of a variety of immune cells, including the functions of B cells, monocytes, and T cells (Supplementary Figure S6). The PPI network of corresponding proteins of the *ERBB2*-related DEGs was screened with the STRING tool with a confidence threshold of 0.4. In total, 84 nodes and 127 edges were connected, and the networks with nodes ≥3 were displayed with Cytoscape (Supplementary Figure S7A). The core network was analyzed with the Cytoscape-MCODE function, showing the networks with an MCODE score of 4.815 (Supplementary Figure S7B) and 4.612 (Supplementary Figure S7C); the proteins of *NPY* and *ESR1* were located in the center of the PPI network (Supplementary Figure S7).

## Discussion

Despite *ERBB2* being a well-known oncogene and regarded as a crucial diagnostic/prognostic biomarker and therapeutic target in multiple tumors, such as breast cancer (Wynn and Tang, 2022), the exact function of *ERBB2* in tumors remains largely unknown. Only a few data are available in PTC, showing an 18%–100% rate of positive expression of *ERBB2* protein *via* immunohistochemistry in 43–45 cases (Kremser et al., 2003; Ruggeri et al., 2016). Thus, expression profiles of the *ERBB2* gene in the PTC need to be comprehensively investigated in the context of a large-scale study. In the present study, we took advantage of a large-scale analysis of gene expression profiles in 510 PTC patients in the TCGA database. The bioinformatic analysis adds the knowledge of detailed relations between *ERBB2* expression and the clinical characteristics, prognosis, and immunoenvironment of PTC. Moreover, it is a novel finding that the expression levels of *ERBB2* were significantly correlated to the markers of the immune cells in the PTC tissues, suggesting that ERB2 may play a crucial role in regulating the tumor-immunoenvironment. The present study uncovered the biological and clinical roles of *ERBB2* in PTC, which might help imply novel ERBB2-based management strategies to improve the prognosis of PTC patients.

*ERBB2* is up-regulated in multiple tumors located in breast, bladder, pancreas, ovary, and esophagus, especially in tumors with poor prognostic characteristics; in breast cancer, high expression of *ERBB2* has become one of the hallmarks of poor prognosis (Dawood et al., 2009). Similarly, our study showed that the expression of *ERBB2* in PTC was aberrantly up-regulated. A step further, we found that *ERBB2* expression in patients with bilateral PTCs was higher than that in those with unilateral foci, which may be related to the fact that bilateral PTC is more aggressive and metastatic than the PTC with unilateral foci (Qu et al., 2016). Meanwhile, we found that the expression of *ERBB2* in classic PTC was higher than that in FVPTC, which may be related to the poorer prognosis of PTC than that of FVPTC (Liu et al., 2018). The prognosis analysis in our study uncovered that the high expression of *ERBB2* is a risk factor for poor prognosis in PTC.

The highly expressed *ERBB2* was deemed to participate in cancer progression, promoting tumor proliferation, invasion, and metastasis (Mitri et al., 2012). PTC patients with high expression of *ERBB2* were prone to suffer from distant metastasis (Kremser et al., 2003). In our study, the expression of *ERBB2* was found to be positively correlated with VEGF-associated genes of *VEGFA*, *FLT*, *KDR*, and *FLT4* (Shibuya, 2011), vascular endothelial cell markers of *PECAM1* and *VWF* (Bauer et al., 2015), and vascular support-related genes of *TEK*, *ANGPT1*, *ANGPT2*, *CDH5*, *CLDN5*, and *JAM2* (Oshi et al., 2021). Furthermore, the expression of *ERBB2* was positively correlated with lymph node metastatic signature genes such as *EVA1A*, *TIMP1*, *SERPINA1*, *FAM20A*, *FN1*, *TNC*, and *MXRA8* (Wu et al., 2021), and distant metastatic signature genes of *MMP2*, *PTTG1*, *VEGFC*, *CXCR4*, and *FGF2* (Liang et al., 2011). Besides, *ERBB2* expression was also positively correlated with proliferation-related genes, such as *MKI67*, *PCNA*, and *MCM2* (Juríková et al., 2016). Collectively, highly expressed *ERBB2* is involved in progressive oncological behaviors of PTC, which might be responsible for the poor prognosis.

Interestingly, high expression of *ERBB2* was found to be associated with dysfunction of iodine metabolism of PTC in our study. The expression of *ERBB2* was negatively correlated with *SLC5A8*, *SCL26A4*, and *TPO*, which relate to iodine absorption, transport, and organization. The downregulated *SLC5A8* was associated with impaired resorption of organic iodine (Porra et al., 2005), making it challenging to transport iodine across the basal membrane to cell cytoplasm in thyroid follicular cells (Anekpuritanang et al., 2021). Meanwhile, *TPO* is the crucial protein to organize iodine with the participation of H_2_O_2_, and *TPO* deficiency would prevent iodine from taking part in the organization process (Kessler et al., 2008). Although the expression of *ERBB2* was positively correlated with that of *TSHR*, the role of *TSHR* in radioiodine refractoriness remains debated because the highly expressed *TSHR* is not only associated with high radioiodine uptake (Hou et al., 2010) but also causes fast thyroid tumor growth (Lu et al., 2010). Therefore, the high expression of *ERBB2* is likely to associate with the poor efficacy of radioactive iodine to some extent, and this mechanism needs to be further studied.

In recent years, targeted drugs, including commonly applied receptor tyrosine kinase inhibitors (RTKi) and novel MEKi, have become valuable antitumor drugs for advanced or progressive thyroid cancer (Jin et al., 2018). Notwithstanding the broad application of RTKi, the occurrence of RTKi-related severe adverse events is frequent, and drug resistance eventually develops in almost all PTC patients (Cheng et al., 2020). Compared to RTKi, the MEKi has the additional capability of inducing tumor differentiation, activating immune recognition, and possesses the characteristic of a lower incidence of severe adverse events (Neuzillet et al., 2014), attracting more attentions and expectations in the field of anti-PTC. Still, MEKi resistance is the typical scenario, often calling for a combination of MEKi and other therapeutics; nevertheless, the improvement of efficacy by the combination is generally limited (Shah et al., 2017), requiring a crucial key to solving the most pressing challenge. MEKi resistance has been proven to be closely related to the high expression of *ERBB2* (Sa et al., 2022). For instance, high *ERBB2* expression is one characteristic phenotype of thyroid cancer resistance to MEKi selumetinib (Montero-Conde et al., 2013). Our study further explored the correlation between the expression of *ERBB2* and MEKi resistance genes of *SPRY2*, *SPRY4*, *ETV4*, *ETV5*, *DUSP4*, *DUSP6*, *CCND1*, *EPHA2*, and *EPHA4* (Wagle et al., 2018). In *BRAF* or *RAS* mutant tumor cells, *DUSP4*, *DUSP6*, *SPRY2*, and *SPRY4* tend to be highly expressed, allowing tumors to evade regular MAPK signaling pathway feedback (Pratilas et al., 2009). Other genes, such as *ETV4*, *ETV5*, *CCND1*, *EPHA2*, and *EPHA4,* also play a role in activating the MAPK pathway, promoting tumor resistance to MEKi (Chesnokov et al., 2022). Together with the previous study (Montero-Conde et al., 2013), our study confirmed the relationship between highly expressed *ERBB2* and MEKi resistance in PTC.

Over the past decade, immune-related diagnostic techniques have successfully become new tools in tumor management. For instance, Michael D Farwell et al. conducted a phase I trial of PET/CT scan with ^89^Zr-labeled IAB22M2C minibody targeting CD8^+^ cells, which visualized the biodistribution of tumor-infiltrating CD8^+^ T cells and predicted early response to immunotherapy (Farwell et al., 2021). In the present research, *ERBB2* expression was inversely correlated with the enrichment scores of CD8^+^ T cells. Therefore, radioactive or non-radioactive probes, such as ^68^Ga-labeled anti-ERBB2 Nanobody (Keyaerts et al., 2016) or polyethylene glycol-conjugated anti-ERBB2 peptides targeting ERBB2 protein (Guan et al., 2018) might be of value for predicting the response to immunotherapy in PTCs. In addition, ERBB2-targeted imaging would overcome the false positive uptake of ^89^Zr-labeled IAB22M2C in CD8^+^ normal tissues, such as the bone marrow and lymphnodes.

The finding, *ERBB2* overexpression related to suppressed T cell infiltration in tumors, is not unique to PTC. Liu et al. (2021) also found that *ERBB2* expression was negatively correlated with the infiltration of B cells and CD8^+^ T cells in cutaneous melanoma, similar to our findings. In breast cancer, *ERBB2* is related to low expression of MHC class I surface antigen, causing impaired recognition between tumor cells and CD8^+^ T cells (Herrmann et al., 2004; Seliger and Kiessling, 2013). Besides, the PD-1/PD-L1 antibodies have been used in clinical trials to treat advanced, progressive, or metastatic thyroid cancer. However, the results were unsatisfactory; most patients still have an unfavorable response to PD-1 antibody therapy (Mehnert et al., 2019), related to the limited T-cell infiltration in PTC (Bastman et al., 2016). DEG analysis showed that in PTC samples with high expression of *ERBB2*, *KLK15* and *KLK1* were downregulated. The down-regulated *KLK*s were previously reported to be responsible for impaired immune microenvironment reprogramming during an antitumor immune response (Srinivasan et al., 2022). In our research, KEGG and GO annotation analysis found that the DEGs were mainly associated with the primary immunodeficiency pathway, which is closely related to tumor occurrence and the failure of antitumor immunotherapy (Tangye et al., 2020). Therefore, increasing tumor-infiltrating immune cells by applying bispecific antibodies or adding cytokines, e.g., PD-L1×CD3 bispecific antibody (Yang et al., 2021) or interleukin-17 (Nagaoka et al., 2020), in *ERBB2* highly expressed tumors may be beneficial to improving the response to immunotherapy in PTC patients.

In addition, we found that *NPY* and *ESR1* were located at the center of the *ERBB2*-related PPI network. *NPY* is a pleiotropic gene initially thought to be an endogenous anxiolytic peptide whose expression can be regulated by stress; however, *NPY* has been found to promote the growth and migration of breast cancer cells in recent years (Lin et al., 2021). *ESR1* encodes an estrogen receptor, plays a crucial role in the occurrence and progression of breast and endometrial cancer, and is the main reason for resistance to estrogen suppression therapy (Piscuoglio et al., 2018). These results suggest that *ERBB2* may be involved in the occurrence and progression of PTC together with *NPY* and *ESR1*; the mechanism needs further investigation.

Despite the fact that our data analysis enhanced the understanding of the roles of *ERBB2* associated with PTC progression, there remain some limitations. First, the role of *ERBB2* in follicular thyroid cancer, poorly differentiated thyroid cancer, and anaplastic thyroid cancer could not be investigated because those pathological types or endpoint information were lacking in online databases. Second, despite large sample studies in public databases prone to possess the superiority of strong evidence, public databases remain to lack some clinical information on PTC tissue around the prognostic tumor status of radioiodine uptake, angiogenesis, proliferation, drug resistance, and serum thyroglobulin levels; Therefore, we cannot perform a clinically based analysis comparing those prognostic factors above, and directly investigating their relations with *ERBB2* expression. Thus, a more comprehensive prospective study is of value in the future to detail the role of *ERBB2* in thyroid cancer. Lastly, this research mainly relied on mRNA data from the TCGA database; therefore, it is necessary to further experimentally investigate the direct evidence of *ERBB2*-related mechanisms in PTC tumorigenesis and progression *in vitro* or *in vivo*.

Because of the complex interaction between *ERBB2* and mutant genes, we didn't separate poor prognosis contributed by *ERBB2* overexpression and gene mutations. To step further, we have planned a more profound analysis in the subsequent study to separate their contributions to poor prognosis. As is known, *BRAF* mutation is a driver gene for tumorigenesis, which causes *ERBB2* overexpression and poor prognosis of PTC (Kebebew et al., 2007; Caria et al., 2016). It remains unknown whether *ERBB2* overexpression independently contributes to the poor prognosis of PTC, which need further validation.

The present work, the first to document, provides a comprehensive study of the relationship between the *ERBB2* expression and clinical characteristics of a large-scale PTC cohort. We found that *ERBB2* was highly expressed in PTC, and elevated *ERBB2* expression was associated with iodine metabolism dysfunction, tumor proliferation, metastasis, angiogenesis, MEKi resistance, and poor prognosis. Meanwhile, *ERBB2* expression was inversely correlated with the infiltration of immune cells in PTC tissues. It seems that *ERBB2* might be a prognostic biomarker and an immunotherapeutic target in PTC, warranting further clinical validation. The study might also be a new start to expect future investigations on the latent mechanisms that bridge *ERBB2* expression, clinical characteristics, and immunosuppressive environment in PTC.

## Data Availability

The original contributions presented in the study are included in the article/Supplementary Material, further inquiries can be directed to the corresponding author.
