# Supplementary material for: ERBB2 as a prognostic biomarker correlates with immune infiltrates in papillary thyroid cancer
Source: Front Genet. 2022 Nov 9;13:966365. doi: 10.3389/fgene.2022.966365 (PMC9682178; doi:10.3389/fgene.2022.966365)
Supplement: Supplementary file 1 [file DataSheet1.PDF]

Supplementary Material

Supplementary Tables

Supplementary Table 1. Characteristics of analyzed papillary thyroid cancer patients.

Characteristic	levels	Low expression of *ERBB2*	High expression of *ERBB2*	*p*
n		255	255	
Genetic alterations	*BRAF* mutation	115 (45.1%)	169 (66.3%)	< 0.001
	KRAS mutation	2 (0.8%)	2 (0.8%)	
	HRAS mutation	7 (2.7%)	9 (3.5%)	
	NRAS mutation	28 (11.0%)	9 (3.5%)	
	Others	103 (40.4%)	66 (25.9%)	
T stage, n (%)	T1	74 (29%)	69 (27.1%)	0.024
	T2	71 (27.8%)	96 (37.6%)	
	T3	91 (35.7%)	84 (32.9%)	
	T4	17 (6.7%)	6 (2.4%)	
	Tx	2 (0.8%)	0 (0.0%)	
N stage, n (%)	N0	115 (45.1%)	114 (44.7%)	1.000
	N1	116 (45.5%)	115 (45.1%)	
	Nx	24 (9.4%)	26 (10.2%)	
M stage, n (%)	M0	140 (54.9%)	146 (57.3%)	0.503
	M1	3 (1.2%)	6 (2.4%)	
	Mx	112 (43.9%)	103 (40.4%)	
Pathologic stage, n (%)	Stage I	137 (53.7%)	149 (58.4%)	0.617
	Stage II	26 (10.2%)	26 (10.2%)	
	Stage III	60 (23.5%)	53 (20.8%)	
	Stage IV	32 (12.5%)	25 (9.8%)	
	Not evaluable	0 (0.0%)	2 (0.8%)	
Gender, n (%)	Female	184 (36.1%)	187 (73.3%)	0.842
	Male	71 (13.9%)	68 (26.7%)	
Race, n (%)	Asian	24 (9.4%)	27 (10.6%)	0.534
	Black or African American	10 (3.9%)	17 (6.7%)	
	White	162 (63.5%)	174 (68.2%)	
	Not report	59 (23.1%)	37 (14.5%)	
Age, n (%)	≤ 45	111 (43.5%)	130 (51.0%)	0.110
	> 45	144 (56.5%)	125 (49.0%)	
Histological type, n (%)	Classical PTC	161 (63.1%)	203 (79.6%)	< 0.001
	Follicular variant PTC	70 (27.5%)	31 (12.2%)	
	Tall cell variant PTC	20 (7.8%)	16 (6.3%)	
	Other types	4 (1.6%)	5 (2%)	
Residual tumor, n (%)	R0	190 (74.5%)	200 (78.4%)	0.109
	R1	29 (11.4%)	25 (9.8%)	
	R2	4 (1.6%)	0 (0%)	
	Not report	32 (12.5%)	30 (11.8%)	
Extrathyroidal extension, n (%)	No	163 (63.9%)	175 (68.6%)	0.349
	Yes	82 (32.2%)	72 (28.2%)	
	Not report	10 (3.9%)	8 (3.1%)	
Primary neoplasm focus type, n (%)	Multifocal	106 (41.6%)	127 (49.8%)	0.073
	Unifocal	144 (56.5%)	123 (48.2%)	
	Not report	5 (2%)	5 (2%)	
Neoplasm location, n (%)	Bilateral	30 (11.8%)	58 (11.5%)	< 0.001
	Isthmus	7 (2.7%)	15 (5.9%)	
	Left lobe	83 (32.5%)	94 (36.9%)	
	Right lobe	132 (51.8%)	85 (33.3%)	
	Not report	3 (1.2%)	3 (1.2%)	
Thyroid disorder history, n (%)	Lymphocytic Thyroiditis	36 (14.1%)	38 (14.9%)	0.469

Characteristic	levels	Low expression of *ERBB2*	High expression of *ERBB2*	*p*
	Nodular Hyperplasia	36 (14.1%)	32 (12.5%)	
	Normal	127 (49.8%)	158 (62.0%)	
	Other thyroid disorders	14 (5.5%)	11 (4.3%)	
	Not report	42 (16.5%)	16 (6.3%)	
PFI event, n (%)	Stable	230 (90.2%)	226 (88.6%)	0.666
	Progress	25 (9.8%)	29 (11.4%)	
OS event, n (%)	Alive	246 (48.2%)	248 (48.6%)	0.799
	Dead	9 (1.8%)	7 (1.4%)	

PTC, papillary thyroid cancer; PFI, progression-free interval; OS, overall survival.

Supplementary Table 2. Correlation analysis between the ERBB2 expression and the scores of tumor-infiltrating immune cells.

Variables	r	*p*
ERBB2-aDC	-0.084	0.058
ERBB2-B cells	-0.176	<0.001
ERBB2-CD8+ T cells	-0.160	<0.001
ERBB2-Cytotoxic cells	-0.219	<0.001
ERBB2-DC	-0.006	0.894
ERBB2-Eosinophils	0.277	<0.001
ERBB2-iDC	0.058	0.190
ERBB2-Macrophages	0.032	0.464
ERBB2-Mast cells	0.011	0.812
ERBB2-Neutrophils	-0.000	0.998
ERBB2-NK CD56bright cells	-0.001	0.986
ERBB2-NK CD56dim cells	-0.218	<0.001
ERBB2-NK cells	0.317	<0.001
ERBB2-pDC	-0.267	<0.001
ERBB2-T cells	-0.164	<0.001
ERBB2-T helper cells	0.058	0.192
ERBB2-Tcm	0.127	0.004
ERBB2-Tem	-0.028	0.531
ERBB2-TFH	-0.111	0.012
ERBB2-Tgd	-0.105	0.017
ERBB2-Th1 cells	0.033	0.454
ERBB2-Th17 cells	0.012	0.791
ERBB2-Th2 cells	-0.055	0.215
ERBB2-TReg	-0.125	0.005

DC, dendritic cells; aDC, activated dendritic cells; pDC, plasmacytoid dendritic cells; iDC, interdigitating dendritic cells; Tcm, central memory T cells; Tem, effector memory T cells; TFH, follicular helper T cells; Tgd, gamma delta T cells; Th1, T helper type 1; Th17, T helper type 17; Th2, T helper type 2; TReg, regulatory T cells; NK, natural killer; CD56bright, cell subset with high cell surface density of CD56; CD56dim, cell subset with low cell surface density of CD56.

Supplementary Table 3. Differentially expressed genes between the tumor samples with high- and low-expression of ERBB2.

Gene name	Base mean	log_2(Fold Change)	LogFcSE	Stat	*p*-value	*p*-adjusted	Regulation
KLK15	37.5640583	-4.1562106	0.22760945	-18.260273	1.7143E-74	3.3011E-70	Down
ARSF	14.7965675	-2.9190891	0.27439334	-10.638338	1.9762E-26	4.4508E-24	Down
CSRP3	1.13922519	-2.6848347	0.44906075	-5.9787785	2.2482E-09	2.7099E-08	Down
KLK1	188.058226	-2.5910716	0.19800316	-13.086011	3.9586E-39	3.909E-36	Down
FGF21	0.82789051	-2.5872935	0.39855072	-6.4917547	8.4842E-11	1.3631E-09	Down
CARTPT	395.861233	-2.5596996	0.34610349	-7.3957636	1.406E-13	3.938E-12	Down
CAMP	64.4133363	-2.3494828	0.15293126	-15.362999	2.8985E-53	6.2015E-50	Down
AQP12B	0.89813002	-2.345725	0.47689329	-4.9187629	8.7093E-07	6.0862E-06	Down
TRIM50	24.3250025	-2.3148399	0.18663161	-12.403257	2.5091E-35	1.6953E-32	Down
PVALB	43.5486158	-2.3030659	0.19371268	-11.889082	1.3488E-32	6.7462E-30	Down
DEFB1	63.1296018	-2.2969402	0.16527066	-13.898052	6.5089E-44	8.9525E-41	Down
CUX2	380.491679	-2.2833029	0.25818688	-8.8436056	9.2678E-19	6.549E-17	Down
KCNA2	41.2885103	-2.2354583	0.1812022	-12.336816	5.738E-35	3.7455E-32	Down
IGLL5	3783.95114	-2.1134815	0.25723298	-8.2162152	2.1003E-16	9.8043E-15	Down
HCN2	49.9340216	-2.1045085	0.1556318	-13.522355	1.1542E-41	1.347E-38	Down
MT3	42.7323922	-2.0901171	0.16247898	-12.863923	7.1836E-38	5.7637E-35	Down
IGLL1	9.42765134	-2.0508377	0.20535165	-9.9869551	1.7384E-23	2.5075E-21	Down
PIP	9.43876187	-2.0452864	0.25869706	-7.906106	2.6556E-15	1.0066E-13	Down
ALDH1L1	279.353381	-2.0400757	0.15736053	-12.964342	1.949E-38	1.7059E-35	Down
SH3GL2	145.501094	-2.0082768	0.19599323	-10.246664	1.225E-24	2.1542E-22	Down
LHX2	8.2029216	-1.9976504	0.18527079	-10.782328	4.1719E-27	9.9795E-25	Down
SLC6A17	77.5265084	-1.9910054	0.17205525	-11.571895	5.7205E-31	2.2031E-28	Down
TERT	5.10480515	-1.9578857	0.33890761	-5.7770486	7.6022E-09	8.1759E-08	Down
KRT85	8.8406546	-1.9433595	0.23533024	-8.2580098	1.4812E-16	7.1664E-15	Down
PAX5	108.427328	-1.9336043	0.24458545	-7.9056393	2.6656E-15	1.0094E-13	Down
MZB1	1258.47141	-1.9309767	0.24029952	-8.0357078	9.304E-16	3.8446E-14	Down
SMR3B	37.753188	-1.8951625	0.33309891	-5.6894888	1.2742E-08	1.3135E-07	Down
TCL1A	57.7556825	-1.8907261	0.25776428	-7.3350975	2.2156E-13	6.0132E-12	Down
BFSP2	19.8962173	-1.886887	0.16075139	-11.73792	8.1466E-32	3.5252E-29	Down
CD79A	1163.31473	-1.8523949	0.23109342	-8.0157838	1.0944E-15	4.4647E-14	Down
AC136428.1	19.6524671	-1.8483093	0.34004157	-5.4355393	5.4631E-08	4.9156E-07	Down
ATP2B3	2.79654805	-1.8321083	0.29010571	-6.3153128	2.6962E-10	3.905E-09	Down
CCL19	771.306638	-1.8229324	0.24298566	-7.5022221	6.2745E-14	1.8732E-12	Down
NWD2	8.5423572	-1.8183403	0.28350584	-6.4137664	1.4197E-10	2.1791E-09	Down
SERPINA9	10.8590516	-1.8046518	0.34016604	-5.3052088	1.1254E-07	9.4884E-07	Down
CALN1	10.7670195	-1.7965257	0.18303505	-9.8152005	9.6845E-23	1.2309E-20	Down
LGALS13	7.19543635	-1.7694555	0.37304	-4.7433399	2.1022E-06	1.3357E-05	Down
FBP2	1.41718449	-1.7674622	0.33711704	-5.2428741	1.5809E-07	1.291E-06	Down
NPY4R	1.58943583	-1.7648993	0.31970853	-5.5203386	3.3835E-08	3.2039E-07	Down
GPRC6A	0.8784237	-1.7546794	0.30107041	-5.8281363	5.605E-09	6.2243E-08	Down
SFTPC	78.6759775	-1.7263094	0.18740425	-9.2116877	3.2104E-20	2.7846E-18	Down
ACOD1	0.68067061	-1.7190331	0.32916327	-5.2224329	1.7659E-07	1.426E-06	Down
SLC22A24	0.82877683	-1.7179706	0.37023798	-4.6401791	3.4811E-06	2.1043E-05	Down
OR2I1P	66.6202213	-1.709728	0.22388105	-7.6367698	2.2274E-14	7.2881E-13	Down
SERPINA3	3.1000149	-1.7067512	0.24920064	-6.8489038	7.4418E-12	1.4896E-10	Down
UBD	119.948928	-1.7041392	0.21130819	-8.0647099	7.341E-16	3.0966E-14	Down
MTRNR2L10	11.6945846	-1.7037149	0.12899453	-13.207652	7.9258E-40	8.2497E-37	Down
DNTT	11.0826581	-1.6975703	0.22572988	-7.5203616	5.4625E-14	1.65E-12	Down
TNFRSF17	99.8144805	-1.6936239	0.22425885	-7.5520939	4.2832E-14	1.3154E-12	Down
FCER2	44.4005184	-1.6905522	0.21782675	-7.7609946	8.4266E-15	2.9664E-13	Down
C8A	29.0254171	-1.6853696	0.22862617	-7.3717266	1.6843E-13	4.6566E-12	Down
NUPR1	11918.7123	-1.6785482	0.12270274	-13.679795	1.3407E-42	1.7805E-39	Down
COL9A3	2543.24678	-1.6784984	0.21708417	-7.7320164	1.0586E-14	3.6565E-13	Down
ETNPPL	61.1645983	-1.6565461	0.23242387	-7.1272631	1.0238E-12	2.4415E-11	Down
IL37	10.9084387	-1.6553098	0.28973478	-5.7131898	1.1088E-08	1.1575E-07	Down
FCRL1	34.5764016	-1.6543741	0.23780189	-6.9569426	3.4774E-12	7.4732E-11	Down
MTRNR2L3	6.34055604	-1.6354945	0.13630955	-11.998385	3.623E-33	1.9932E-30	Down
HHATL	4809.15268	-1.6297323	0.15237801	-10.695325	1.0704E-26	2.5137E-24	Down
ATP1A3	35.2398008	-1.6247357	0.14054245	-11.560463	6.5356E-31	2.4921E-28	Down
BRS3	1.62013012	-1.6232838	0.28968913	-5.6035371	2.1002E-08	2.0765E-07	Down
CSAG1	15.400073	-1.6006945	0.22052551	-7.2585458	3.9127E-13	1.0093E-11	Down
MTRNR2L13	0.91778711	-1.5997284	0.23856801	-6.7055445	2.0066E-11	3.652E-10	Down
NXNL1	2.12925878	-1.5993573	0.1855672	-8.6187501	6.7689E-18	4.1117E-16	Down
CR2	308.128529	-1.5953659	0.21588638	-7.3898406	1.47E-13	4.1025E-12	Down
ISL2	4.04026605	-1.5946391	0.22484552	-7.0921541	1.3204E-12	3.0744E-11	Down

CHGB	1170.78678	-1.5807278	0.19206807	-8.2300395	1.8715E-16	8.8654E-15	Down
ADIPOQ	6.50232148	-1.5740432	0.38405829	-4.0984486	4.1593E-05	0.00019601	Down
GSDMC	41.6589934	-1.5723361	0.13649861	-11.519063	1.0575E-30	3.8423E-28	Down
CACNG6	2.4950039	-1.5717106	0.28182294	-5.5769436	2.4478E-08	2.3842E-07	Down
PRAMEF17	2.14399866	-1.571019	0.30250082	-5.1934372	2.0645E-07	1.6516E-06	Down
KRT71	4.21776141	-1.5673818	0.21950838	-7.1404193	9.3047E-13	2.2424E-11	Down
NEB	884.587906	-1.5614897	0.18956943	-8.2370333	1.7653E-16	8.4142E-15	Down
RTP5	5.69646592	-1.5594382	0.23031721	-6.7708279	1.2805E-11	2.4449E-10	Down
MTRNR2L11	1.18159013	-1.5547795	0.22551874	-6.8942366	5.4155E-12	1.1201E-10	Down
AICDA	8.67324021	-1.5542236	0.26590993	-5.8449251	5.068E-09	5.6903E-08	Down
SYNPR	130.67309	-1.5526165	0.22082401	-7.031013	2.0504E-12	4.591E-11	Down
FDCSP	166.899986	-1.5483761	0.42234146	-3.6661712	0.00024621	0.00096235	Down
RALYL	0.74305168	-1.5451994	0.3189093	-4.8452629	1.2644E-06	8.5044E-06	Down
AMPD1	32.1408618	-1.544537	0.27232932	-5.6715779	1.4149E-08	1.4461E-07	Down
MTRNR2L6	7.7321332	-1.5438181	0.13529653	-11.410626	3.7006E-30	1.1976E-27	Down
C14orf180	4.93483926	-1.5352432	0.26453312	-5.8035956	6.4908E-09	7.1013E-08	Down
PRRT4	119.155417	-1.534678	0.2014125	-7.6195771	2.5451E-14	8.2159E-13	Down
TBX5	33.5379982	-1.5343514	0.23627328	-6.4939695	8.3604E-11	1.3472E-09	Down
FCRLA	115.808051	-1.5249124	0.24461198	-6.2340054	4.5466E-10	6.3076E-09	Down
ACSL6	58.5307445	-1.5081484	0.1450514	-10.397338	2.5496E-25	4.7897E-23	Down
C4orf51	1.44765987	-1.5028935	0.20060294	-7.4918819	6.7893E-14	2.0175E-12	Down
CXCL11	210.963901	-1.5010794	0.17825914	-8.4207707	3.7402E-17	2.0006E-15	Down
LAMP5	1370.73066	1.55231199	0.18951963	8.19077156	2.5956E-16	1.1872E-14	UP
ASTL	10.13107	1.55888947	0.18880019	8.25682156	1.496E-16	7.229E-15	UP
KRT4	5.19133942	1.57694341	0.24280797	6.49461143	8.3248E-11	1.342E-09	UP
ESR1	1695.51418	1.57734776	0.1360843	11.590961	4.5797E-31	1.7815E-28	UP
FGF4	1.54012731	1.58848992	0.26845091	5.9172455	3.2738E-09	3.8142E-08	UP
CGA	3.33863378	1.6063682	0.25160627	6.38445216	1.7201E-10	2.5925E-09	UP
GRM4	542.861215	1.68404489	0.17556189	9.59231465	8.6131E-22	9.3438E-20	UP
TGM4	8.23666708	1.71771955	0.17060111	10.0686304	7.6029E-24	1.1903E-21	UP
TRPC5	1005.30379	1.72342676	0.19897065	8.66171355	4.6472E-18	2.9054E-16	UP
LCN10	350.454897	1.73201442	0.17365611	9.97381776	1.9845E-23	2.8412E-21	UP
ZNF648	3.1163149	1.7379652	0.21770294	7.98319565	1.4259E-15	5.7144E-14	UP
LGALS14	1.06933578	1.76229368	0.28157562	6.25868701	3.8823E-10	5.4568E-09	UP
FREM3	10.4074695	1.7704684	0.15451341	11.4583482	2.1355E-30	7.2142E-28	UP
GLP1R	127.974044	1.77088414	0.17681329	10.0155602	1.3022E-23	1.9363E-21	UP
ERICH3	42.9523117	1.77711295	0.19599454	9.0671554	1.2216E-19	9.7813E-18	UP
GDF6	61.5031428	1.78673024	0.19438008	9.19194111	3.8582E-20	3.3092E-18	UP
LCE2C	0.44011634	1.79939459	0.42899963	4.19439664	2.736E-05	0.00013433	UP
KLK6	345.519276	1.79964374	0.2478152	7.26203931	3.813E-13	9.8686E-12	UP
ENTPD2	454.993694	1.80317462	0.14742478	12.2311501	2.1192E-34	1.2752E-31	UP
SLCO1B3-SLCO1B7	0.78969346	1.80731768	0.33024335	5.47268452	4.4327E-08	4.0821E-07	UP
SHISA6	818.103279	1.83799412	0.15784167	11.6445429	2.4463E-31	1.013E-28	UP
DMRTB1	15.4332307	1.85628176	0.31048743	5.97860521	2.2506E-09	2.7119E-08	UP
MC5R	1.41738132	1.86679882	0.21772778	8.5740038	9.9948E-18	5.8946E-16	UP
MEGF11	66.9941325	1.86745279	0.1719945	10.857631	1.8345E-27	4.648E-25	UP
AL355987.1	3.70961725	1.88779943	0.34992747	5.39483062	6.8588E-08	6.0335E-07	UP
B3GNT6	7.93927337	1.89268614	0.1989323	9.51422215	1.8308E-21	1.8953E-19	UP
APOC3	1.14158812	1.89480873	0.40890569	4.63385271	3.5892E-06	2.1602E-05	UP
LCN15	2.01945199	1.90917576	0.24039846	7.94171391	1.9941E-15	7.8203E-14	UP
ALPP	3.05745375	2.03322037	0.28875271	7.04138961	1.9033E-12	4.2966E-11	UP
CACNA1E	37.0548361	2.05230331	0.18487368	11.1011117	1.2389E-28	3.5344E-26	UP
LCN6	557.97469	2.06257082	0.18989352	10.8617229	1.7541E-27	4.5035E-25	UP
NETO1	181.769819	2.07261789	0.17876518	11.5940808	4.4158E-31	1.7353E-28	UP
PRLHR	13.6506303	2.0809739	0.31529473	6.60009107	4.1091E-11	7.0021E-10	UP
TRHR	18.74767	2.10491517	0.20066599	10.4896457	9.6389E-26	1.9334E-23	UP
CYP2W1	56.8601844	2.1192601	0.1874716	11.3044328	1.2476E-29	3.8132E-27	UP
FGFBP1	562.845495	2.19951586	0.19992049	11.0019533	3.7394E-28	1.0001E-25	UP
HS3ST4	11.6775496	2.23015287	0.20237727	11.0197792	3.0681E-28	8.2629E-26	UP
SLC30A3	486.113453	2.24514659	0.19011613	11.8093433	3.4928E-32	1.6207E-29	UP
SRRM4	36.6495118	2.25373043	0.21568462	10.4491941	1.4778E-25	2.8889E-23	UP
C16orf82	1.12142703	2.25914001	0.34426438	6.56222416	5.3011E-11	8.8764E-10	UP
LIX1	596.783829	2.29163939	0.16788096	13.6503833	2.0082E-42	2.4948E-39	UP
VGF	158.167397	2.30464896	0.20866909	11.0445153	2.3302E-28	6.3646E-26	UP
PRMT8	479.752528	2.30701079	0.21072648	10.947892	6.8012E-28	1.7818E-25	UP
NPY	37.8932894	2.32478263	0.24971538	9.30972952	1.2816E-20	1.1893E-18	UP
GSX2	7.73433797	2.33193448	0.29161723	7.99655922	1.2794E-15	5.1596E-14	UP
INMT-MINDY4	8.48018403	2.33597959	0.20695667	11.2872881	1.5164E-29	4.5626E-27	UP
RBFOX1	183.149916	2.3623115	0.22544818	10.4782902	1.0869E-25	2.1577E-23	UP
GAP43	736.523619	2.3812451	0.20809577	11.4430252	2.5484E-30	8.5343E-28	UP
GMNC	9.7825676	2.38187697	0.25146147	9.47213502	2.7418E-21	2.7788E-19	UP
MYRF	605.29484	2.39198928	0.14954197	15.9954373	1.3749E-57	3.7821E-54	UP

INMT	4675.61194	2.44833546	0.15528202	15.7670253	5.2463E-56	1.2628E-52	UP
ELFN2	328.734026	2.69703659	0.19756394	13.6514617	1.9787E-42	2.4948E-39	UP
NKAIN3	3.33022066	2.70148043	0.32233872	8.38087477	5.2536E-17	2.7049E-15	UP
NYAP2	40.5256495	3.02659618	0.23851742	12.6892041	6.7872E-37	5.2278E-34	UP
TAGLN3	109.361098	3.05986335	0.18207117	16.8058645	2.2107E-63	1.4189E-59	UP
RSPO1	97.1688114	3.2335363	0.27374185	11.8123564	3.3698E-32	1.5827E-29	UP
SPAG11A	1.37789859	3.35317392	0.42973004	7.8029777	6.0463E-15	2.1803E-13	UP
SPAG11B	2.10727301	3.69363905	0.38118848	9.68979726	3.3319E-22	3.819E-20	UP
GLRA1	6.07665372	3.73370034	0.28352132	13.1690286	1.3229E-39	1.3407E-36	UP

Supplementary Figures

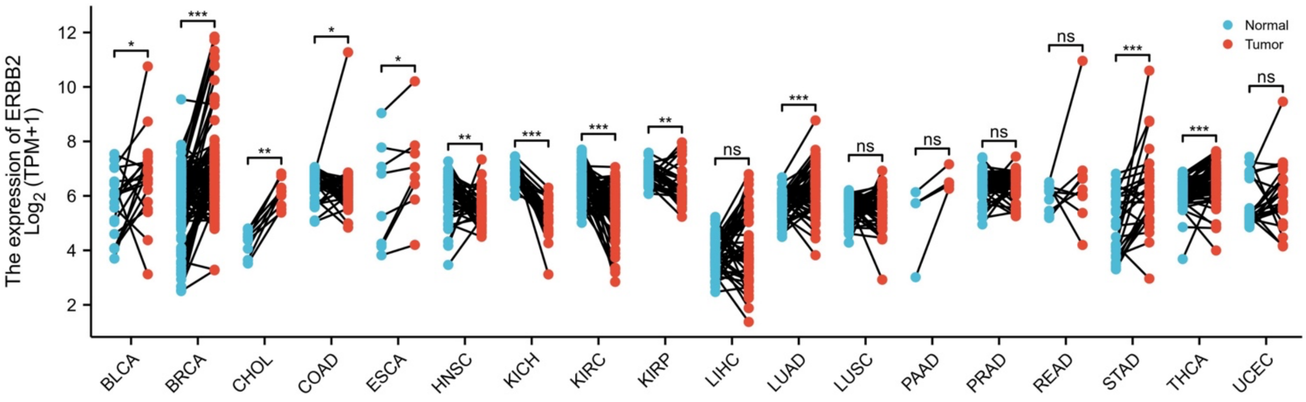

Supplementary Figure 1. The mRNA expression of ERBB2 in pan-cancers and paired adjacent normal samples. Compared with paired normal tissues, mRNA expression of ERBB2 was upregulated in 7 of 18 cancer types: BLCA (p = 0.036), BRCA (p < 0.001), CHOL (p = 0.004), ESCA (p = 0.016), LUAD p < 0.001), STAD (p < 0.001), and THCA (p < 0.001). BLCA, bladder urothelial carcinoma; BRCA, breast invasive carcinoma; CHOL, cholangiocarcinoma; COAD, colon adenocarcinoma; DLBC, lymphoma diffuse large b-cell lymphoma; ESCA, esophageal carcinoma; HNSC, head and neck squamous cell carcinoma; KICH, kidney chromophobe; KIRC, kidney renal clear cell carcinoma; KIRP, kidney renal papillary cell carcinoma; LIHC, liver hepatocellular carcinoma; LUAD, lung adenocarcinoma; LUSC, lung squamous cell carcinoma; PAAD, pancreatic adenocarcinoma; PRAD, prostate adenocarcinoma; READ, rectum adenocarcinoma; STAD, stomach adenocarcinoma; THCA, thyroid carcinoma; UCEC, uterine corpus endometrial carcinoma. $^{*}p < 0.05$, $^{**}p < 0.01$, $^{***}p < 0.001$.

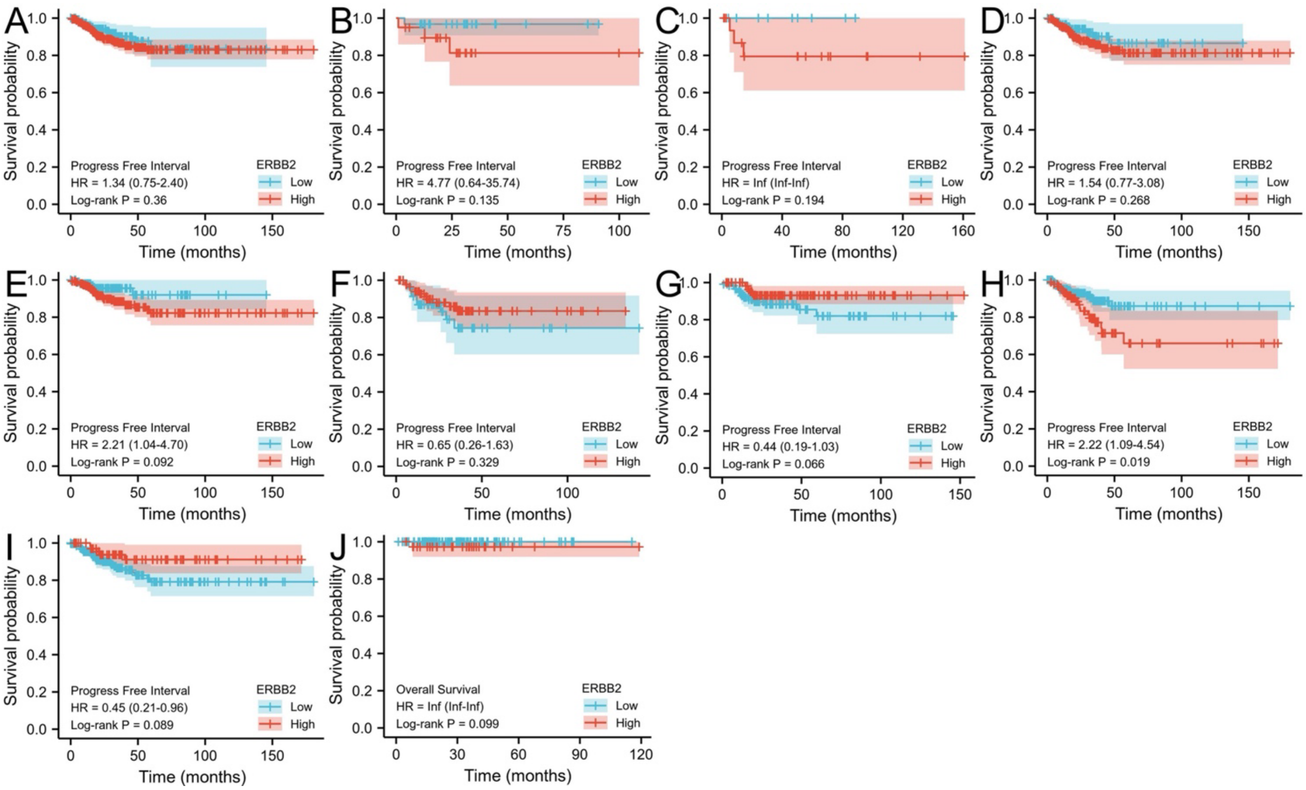

Supplementary Figure 2. Kaplan-Meier curves of progression-free interval comparing high and low expression of *ERBB2* in papillary thyroid cancer patients with anthropologic characteristics and medical history of thyroid disorders: **(A)** total, **(B)** race of Asian, **(C)** race of black or African American, **(D)** race of white, **(E)** female, **(F)** male, **(G)** age ≤ 45 years, **(H)** age > 45 years, **(I)** none of thyroid disorder history, and **(J)** pre-existing thyroid disorders. HR, hazard ratio; Inf, infinity.

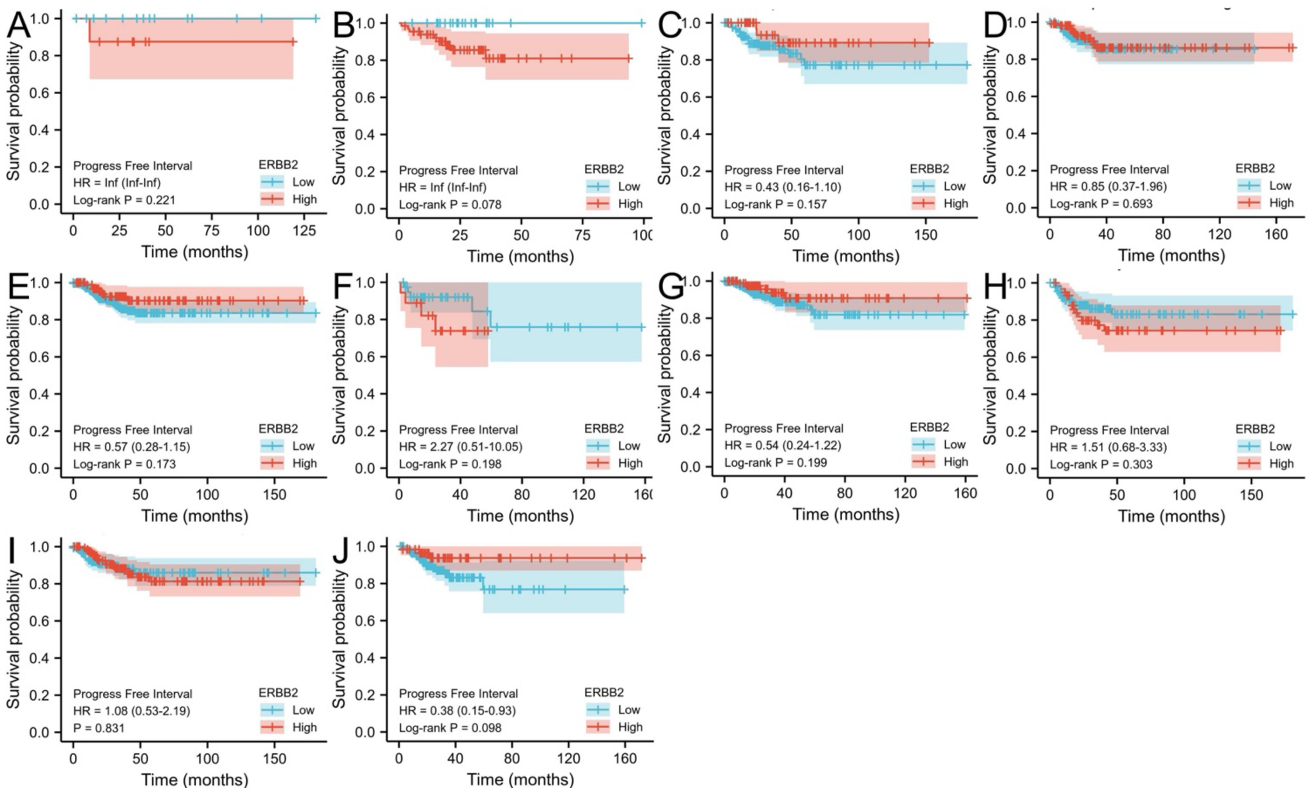

Supplementary Figure 3. Kaplan-Meier curves of progression-free interval comparing high and low expression of *ERBB2* in papillary thyroid cancer patients with divergent tumor locations and extensions: **(A)** tumor located in the isthmus, **(B)** tumor located in bilateral, **(C)** tumor located in the left lobe. **(D)** tumor located in the right lobe, **(E)** residual tumor of R0, **(F)** residual tumor of R1&R2, **(G)** none extrathyroidal extension, **(H)** existing extrathyroidal extension, **(I)** unifocal tumor, and **(J)** multifocal tumor. HR, Hazard ratio; Inf, infinity.

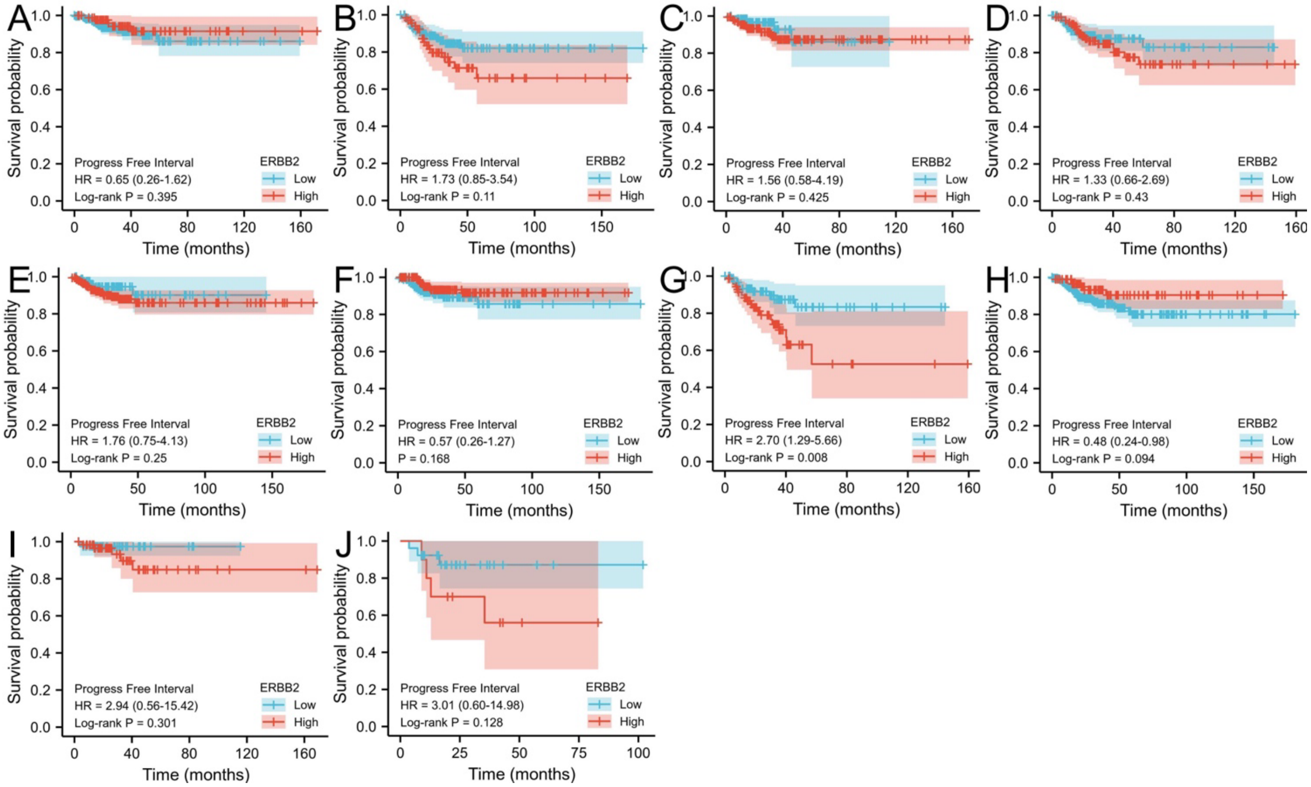

Supplementary Figure 4. Kaplan-Meier curves of progression-free interval comparing high and low expression of *ERBB2* in papillary thyroid cancer patients with clinicopathologic characteristics: **(A)** T stage of T1&T2, **(B)** T stage of T3&T4, **(C)** N stage of N0, **(D)** N stage of N1, **(E)** M stage of M0, **(F)** pathologic stage of I&II, **(G)** pathologic stage of III&IV, **(H)** histological type of classical papillary thyroid cancer; **(I)** histological type of follicular variant papillary thyroid cancer, and **(J)** the histological type of tall cell variant papillary thyroid cancer. HR, Hazard ratio.

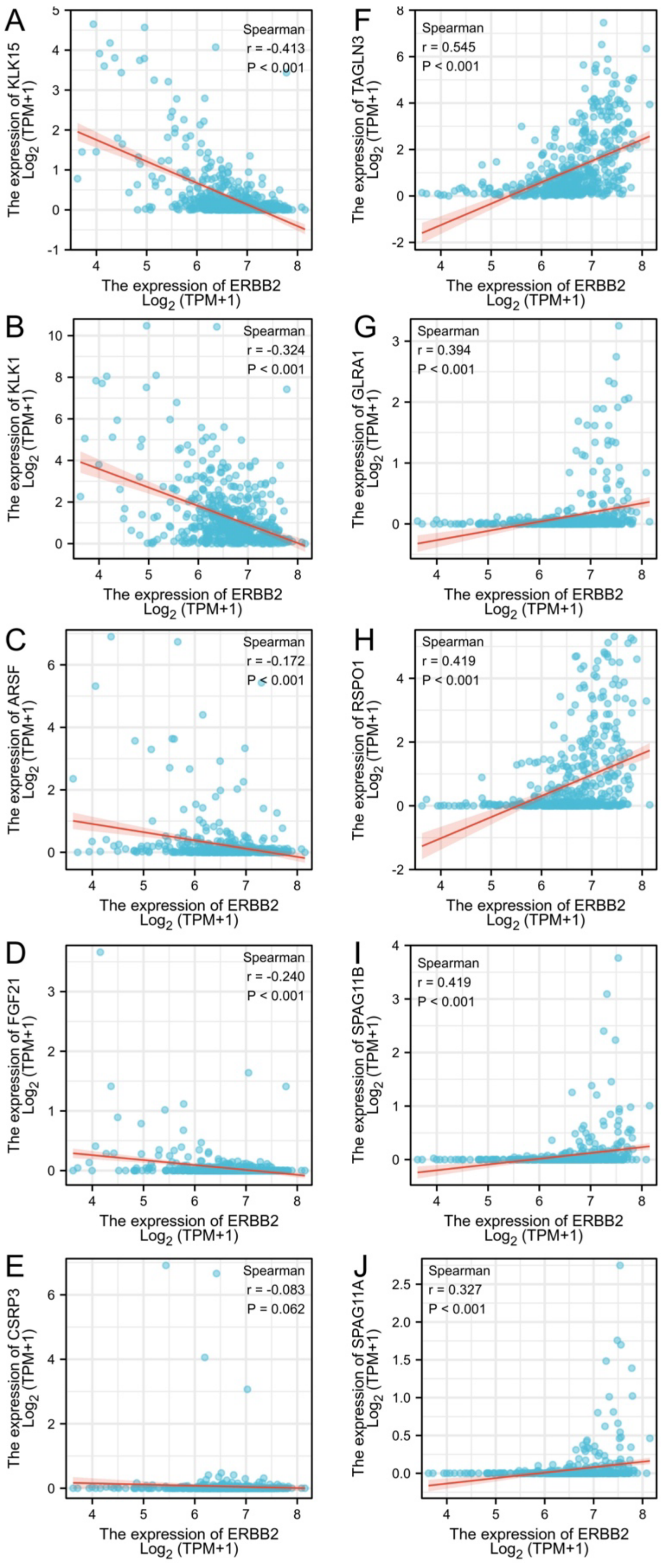

Supplementary Figure 5. The correlation analyses between the expression of *ERBB2* and ten *ERBB2*-related differentially expressed genes (DEGs) in papillary thyroid cancer. **(A–E)** The correlation analyses between the expression of *ERBB2* and five *ERBB2*-related DEGs with down-regulation. **(F–J)** The correlation analyses between the expression of *ERBB2* and five *ERBB2*-related DEGs with up-regulation.

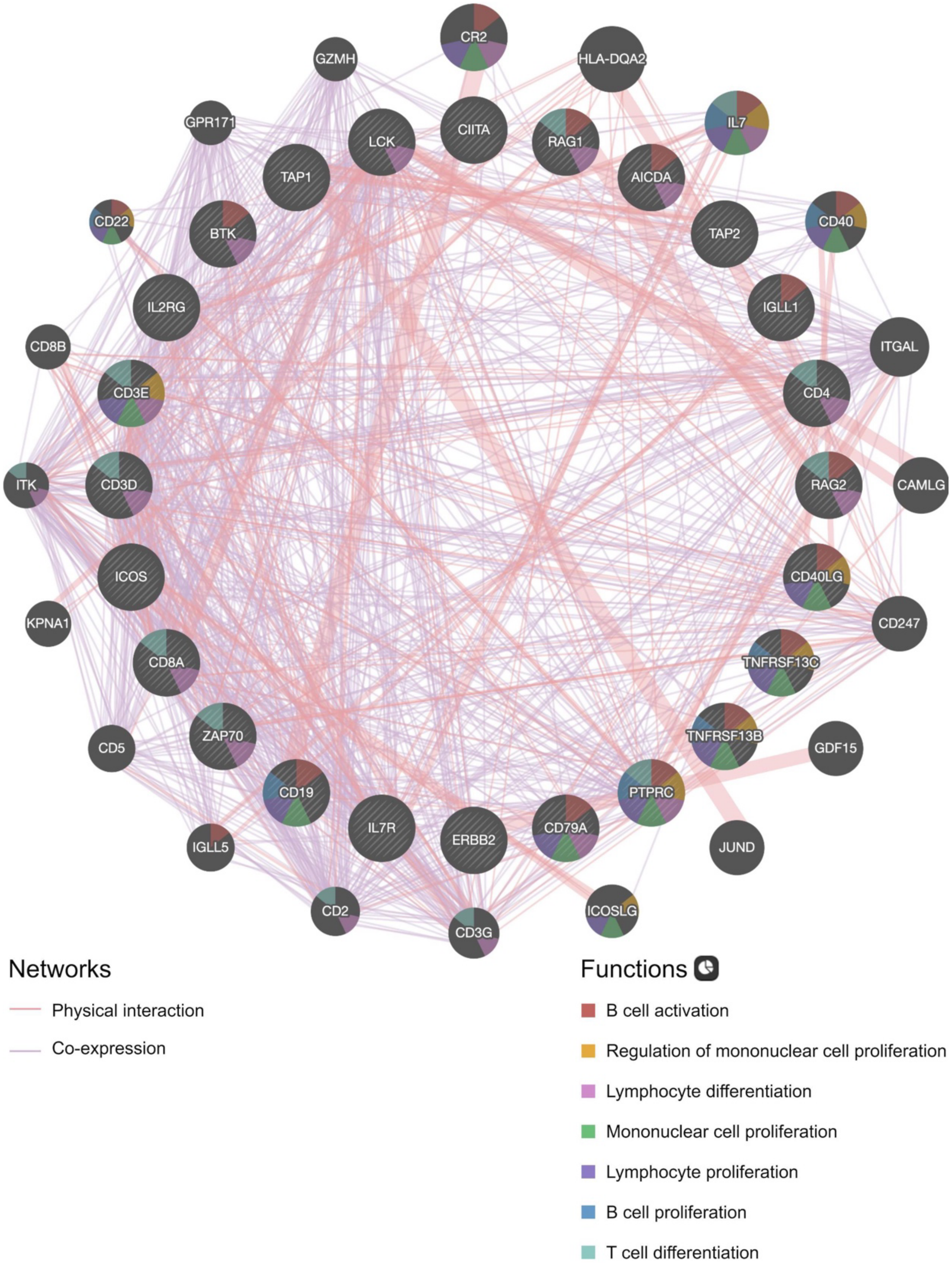

Supplementary Figure 6. The gene-gene interaction network of *ERBB2*-related differentially expressed genes involved in the primary immunodeficiency pathway.

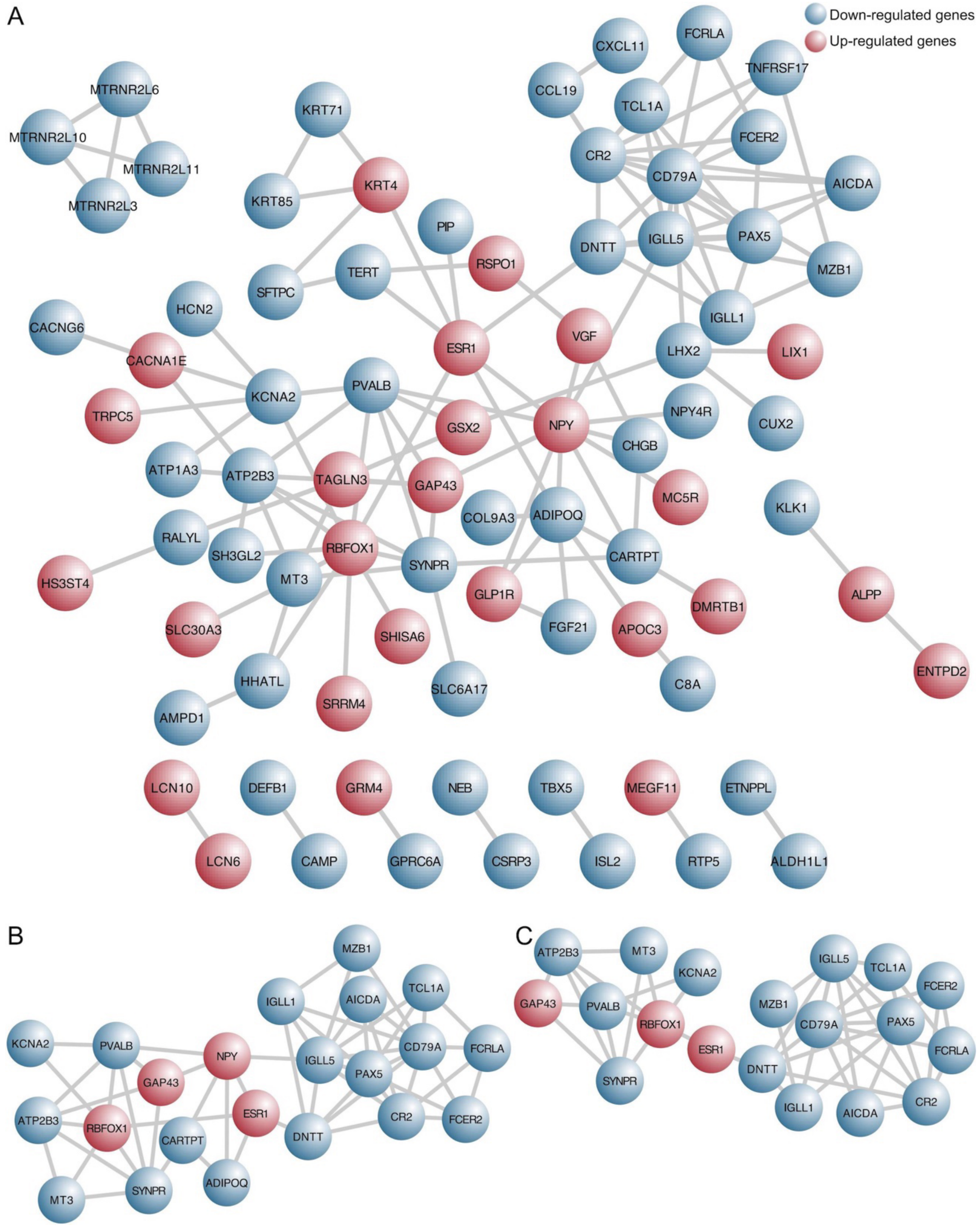

Supplementary Figure 7. The protein-protein interaction (PPI) networks of the corresponding proteins of *ERBB2*-related differentially expressed genes. **(A)** The PPI network of all *ERBB2*-related differentially expressed genes. **(B)** The core PPI network with an MCODE score of 5.333. **(C)** The core PPI network with an MCODE score of 5.222.
